# Educational attainment and HIV testing and counselling service utilisation during antenatal care in Ghana: Analysis of Demographic and Health Surveys

**DOI:** 10.1371/journal.pone.0227576

**Published:** 2020-01-15

**Authors:** Francis Sambah, Linus Baatiema, Francis Appiah, Edward Kwabena Ameyaw, Eugene Budu, Bright Opoku Ahinkorah, Joseph Kojo Oduro, Abdul-Aziz Seidu

**Affiliations:** 1 Department of Health, Physical Education, and Recreation, University of Cape Coast, Cape Coast, Ghana; 2 Department of Population and Health, College of Humanities and Legal Studies, University of Cape Coast, Cape Coast, Ghana; 3 The Australian Centre for Public and Population Health Research, Faculty of Health, University of Technology Sydney, Sydney, New South Wales, Australia; Ohio University College of Health Sciences and Professions, UNITED STATES

## Abstract

**Introduction:**

Receiving results for Human Immunodeficiency Virus (HIV) testing and counselling during antenatal care (ANC) is critical for eliminating mother-to-child transmission. We investigated the educational attainment of women and receiving results for HIV testing and counselling (HTC) during ANC in Ghana.

**Materials and methods:**

We extracted data from the women’s files of the 2008 and 2014 Ghana Demographic and Health Surveys. The study sampled 2,660 women aged 15–49 with complete data on receiving HIV testing results during ANC. We computed the highest educational attainment and receipt of HTC results for each of the surveys and presented it with a dot plot. Two Binary Logistic Regression Models were fitted to determine the likelihood of receiving HTC results by the educational attainment of the women.

**Results:**

We found that receiving HTC results was highest among women with secondary or higher education (87.4% in 2008 and 89.5% in 2014) and least among those with no education (69.9% in 2008 and 76.8 in 2014). From the regression analysis, women with secondary or higher level of education [AOR = 1.535; CI = 1.044, 2.258], richest women [AOR = 5.565; CI = 2.560, 12.10], and women aged 30–34 years [AOR = 1.693; CI = 1.171, 2.952], were more likely to receive HTC results. However, those who did not know that a healthy-looking person can have HIV or not [AOR = 0.322; CI = 0.161, 0.646] were less likely to receive HTC results.

**Conclusion:**

Despite the relatively high receipt of HTC results at ANC observed between 2008 and 2014, our findings revealed disparities driven by educational attainment, wealth status, age, ANC visits and residence. This indicates that women with no education, those from rural areas, younger and poor women are missing out on the full continuum of HTC service at ANC. The Health Promotion Unit of Ghana Health Service through Community Health Nurses and the Community-Based Health Planning and Services, should intensify their education programs on HIV and make full utilisation of HIV testing and counselling service appealing to women during ANC. This is particularly to be prioritised among the least educated, younger women and rural dwellers.

## Introduction

Testing and counselling for Human Immunodeficiency Virus (HIV) during antenatal care (ANC) is critical for eliminating mother-to-child transmission [1]. Mother-to-child transmission (MTCT) or vertical transmission is high on the global scale, ranging between 15% and 45% in the absence of interventions, and underlies most childhood infections [1,2]. The World Health Organisation (WHO) recommends provider-initiated testing and counselling (PITC) at ANC especially in high prevalence settings [3]. Efforts to mitigate MTCT have led to a decline globally, meanwhile, most of these cases occur in sub-Saharan African countries including Ghana. Ghana is one of the twenty-three high priority countries earmarked for PMTCT [2]. Currently, it has 53% coverage of the Prevention of Mother-to-Child Transmission (PMTCT) [4].

In 2016, the Ghana AIDS Commission (GAC) reported a 2.4% national HIV prevalence rate among pregnant women who attended ANC clinics. Significant regional variation was noted with Volta and Brong Ahafo regions leading by 2.7% apiece whilst Northern region recorded the least prevalence (0.7%) [4]. The projections made for 2018 on the number of Acquired Immune Deficiency Syndrome (AIDS) induced deaths among pregnant women (356) and AIDS orphans (178,120) are expected to decline to 176 and 118, 257 respectively by 2022 [4]. In 2017, the number of new infections originating from MTCT was estimated to decline from 2,230 to 1,614 between 2019 and 2022 [4]. The drive to achieve these projections calls for much research attention to explore specific factors that require research directed policy initiatives.

Studies from other sub-Saharan African countries such as Tanzania [5] Congo, Mozambique, Uganda [6] and Ethiopia [7] have indicated that educated pregnant women are likely to fully utilise HIV Testing and Counselling (HTC) service compared to women who are not educated. Mahande et al. [5] explained that higher education offers a better understanding of HIV infection and prevention and as a result may increase the possibility of utilising the full continuum of HTC service. In the case of Ghana, no national-level empirical study has been undertaken to determine whether the level of education contributes to getting results for HTC at ANC or otherwise. This study, therefore, seeks to assess the influence of educational attainment on HTC service utilisation in Ghana (receiving HIV test results during ANC). The outcome of this study will have great benefit for Ghana and other high-priority PMTCT countries by prompting health policy makers to be sensitive to the educational attainment of women when planning on how to achieve universal HTC service utilisation for all pregnant women.

## Materials and methods

### Data source

Data for this study were from the 2008 and 2014 Ghana Demographic and Health Surveys (GDHS) [8, 9]. These surveys cover a wide range of maternal and child health conditions in Ghana. The GDHS form part of the DHS programme which monitors health indicators in over 90 low- and middle-income countries. The DHS programme aims to enhance global understanding of health and population situation in low- and middle-income countries. Collection and dissemination of nationally representative and accurate data on HIV/AIDS, maternal and child health have been part of the core aims of the DHS programme since 1984 [10].

### Inclusion criteria and study sample

We included women between 15 and 49 years with complete data who gave birth in either private or public health facilities prior to the surveys. The datasets from 2008 (n = 586) and 2014 (n = 2,074) resulted in a sample of 2,660. These two surveys were used because these are the only GDHS that investigated HIV testing and counselling and receipt of HIV test results as part of ANC visit.

### Definition of study variables

#### Dependent variable

The dependent variable for the study was whether women obtained results of HIV test as part of ANC visit. All women who indicated that they obtained results of HIV test as part of ANC visit were coded as 1 whilst those who reported that they did not were coded as 0. Counselling is embedded in the HIV testing service such that healthcare providers conduct pre-and post-counselling for HIV testing. Investigating this variable across two consecutive and comparable national surveys is of much essence as Ghana is one of the twenty-three priority countries for the Prevention of Mother to Child Transmission of HIV (PMTCT) [11].

#### Independent variable and covariates

The main independent variable for the study was highest educational attainment of women. The variable originally had four outcomes; no education, primary, secondary and higher. However, this variable was recoded as “No Education”, “Primary”, “Secondary/ Higher”. We combined secondary and higher education because only 4.9% of the women had attained higher education. Included covariates were; age, marital status, residence, wealth status, ANC visits year of survey, whether a healthy-looking person can have HIV or not and region of residence. We recoded marital status into “married” and “not married”, and ANC visits into “below 4 visits”, and “at least 4 visits”.

#### Analytical procedure

**Fig 1:**
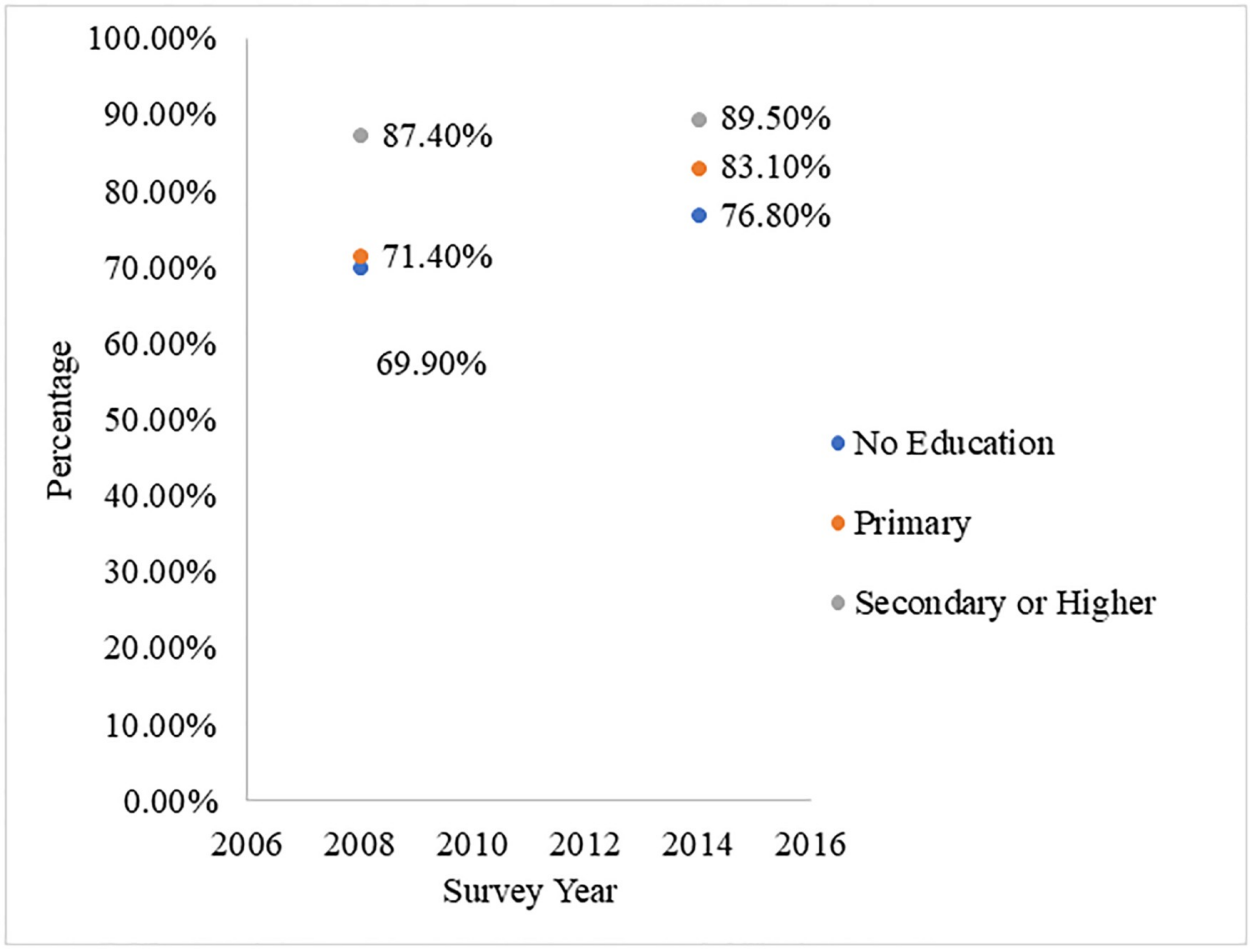
Getting results for HIV testing and counselling at ANC: 2008–2014. 2008 and 2014 GDHS.

We computed the highest educational attainment and obtaining HTC results for each of the surveys. This was presented in a dot plot (Fig 1). Data from the two surveys were pooled together afterwards for further analysis. Using a chi-square test of independence, the association between HTC results, the independent variable and covariates were computed (Table 1). The variables that showed a significant relationship at the initial data exploration at the descriptive level (Table 1) were used to develop Binary Logistic Regression Models. Two sequential Binary Logistic Regression Models were fitted to determine the likelihood of HTC results across the educational attainment of the women (Model 1). The covariates were fitted to ascertain the influence of these on the relationship between education and HTC results (Model 2). Model 1 was presented as Crude Odds Ratio (COR) and Model 2 was presented as Adjusted Odds Ratio (AOR) with their respective confidence intervals (CIs) which were set at 5% margin of error. Sample weights were applied and all analyses were carried out with STATA version 13 and Microsoft Excel.

**Table 1:** Socio-demographic characteristics and getting HTC results during ANC.

	Sample (N = 2,660)		Getting HTC results during ANC		
Variable	Frequency	%	No	Yes	X^2^ (p value)
**Educational attainment**					69.1 (P<0.001)
No Education	524	19.7	24.6	75.4	
Primary	501	18.9	19.7	80.3	
Secondary or Higher	1,635	61.5	11.0	89.0	
**Covariates**					
**Age**					8.8 (P<0.05)
15–19	132	5.0	22.6	77.4	
20–24	496	18.7	18.2	81.9	
25–29	741	27.9	14.8	85.2	
30–34	637	24.0	14.2	85.8	
35–39	469	17.6	15.9	84.1	
40–44	52	5.7	15.8	84.2	
45–49	33	1.2	20.0	80.0	
**Marital Status**					1.14(P = 0.285)
Not married	948	35.6	14.9	85.1	
Married	1,712	64.4	16.4	83.5	
**Residence**					28.5(P<0.001)
Urban	1,400	52.6	12.1	87.9	
Rural	1,260	47.4	19.7	80.3	
**Wealth Status**					97.4(P<0.001)
Poorest	399	15.0	24.3	75.7	
Poorer	476	17.9	21.7	78.3	
Middle	490	18.4	16.5	83.5	
Richer	672	25.3	11.0	89.0	
Richest	624	23.5	4.8	95.2	
**ANC Visits**					2.9 (P<0.05)
Below 4 Visits	205	7.7	20.1	79.9	
At least 4 Visits	2,455	92.3	15.7	84.3	
**Healthy looking person can have HIV**					32.9(P<0.001)
No	286	10.8	18.2	81.8	
Yes	2,295	86.3	14.8	85.2	
Don’t Know	78	2.9	37.1	62.9	
**Region of residence**					101.1 (P<0.001)
Western	243	9.1	17.5	82.5	
Central	323	12.1	17.4	82.6	
Greater Accra	503	18.9	7.2	92.8	
Volta	183	6.9	8.6	91.4	
Eastern	246	9.3	13.7	86.3	
Ashanti	530	19.9	17.4	82.6	
Brong Ahafo	267	10.0	13.3	86.7	
Northern	194	7.3	36.4	63.6	
Upper East	102	3.8	12.2	87.8	
Upper West	69	2.6	20.0	80.0	

GDHS 2008 and 2014

#### Ethical consideration

In each of these surveys, ethical approval was obtained from ICF Macro and Ghana Health Service. We obtained these datasets by applying to Measure DHS which in turn assessed our request and granted us access to use the data. The data can be freely obtained from the website of the DHS Programme through https://dhsprogram.com/what-we-do/survey/survey-display-437.cf.

## Results

### Descriptive results for the study

#### Getting results for HIV testing and counselling at ANC: 2008–2014

As illustrated in Fig 1, there was a general increase in the proportion of women who had received HTC results between 2008 and 2014. However, women with secondary/higher education dominated in both surveys. In 2008 for instance, 87.4% of women with secondary/higher education had received HTC results, whilst 69.9% of those without formal education obtained their results. In the 2014 survey, whereas 89.5% HTC results were obtained by women of secondary/higher education, 76.8% of those without formal education reported the same.

#### Socio-demographic characteristics of women and getting HTC results during ANC

Table 1 shows the results for the socio-demographic characteristics of women and receipt of HTC results at ANC. More than six out of ten had completed secondary or higher education (61.5%). Getting results for HTC was lowest among women who had no formal education (75.4%) but was more prevalent among those with secondary/higher education (89.0%). Women between 25 and 29 years were more (27.9%) with only 1.2% between 45 and 49 years. Most women aged 30–34 had received results for HTC (85.8%). Although most women were married (64.4%), getting results for HTC was highest among those who were not married (85.1%).

More than half of the women were residing in urban locations (52.6%) and most of these urban residents obtained their HTC results during ANC (87.9%). A significant proportion of the women were within the richer wealth quintile (25.3%). Almost all richest women received HTC results (95.2%) followed by those in the richer wealth quintile (89.0%). Nine out of ten of the women had at least 4 antenatal visits (92.3%) and 84.3 percent of these women had their HTC results. Most of the women believed that a healthy-looking person can have HIV (86.3%) and getting HTC results was highest among the same group (62.9%). Ashanti region was accommodating a significant proportion of these women (19.9%), meanwhile, obtaining HTC results dominated among women in the Greater Accra region (92.8%).

#### Binary logistic regression of educational attainment and getting HTC results during ANC

The outcome for the Binary Logistic Regression has been reported in Table 2. At the bivariate level (Model 1), women who had secondary or higher educational attainment were almost three times likely to receive HTC results compared with women who have no formal education [COR = 2.660; CI = 1.984, 3.568]. No much variation occurred after adjusting for the covariates, in Model 2, as the likelihood of receiving HTC results was still high among women having secondary/higher education [AOR = 1.535; CI = 1.044, 2.258]. Women between 30 and 34 years had higher odds of receiving HTC results compared with those aged 15–19 [AOR = 1.693; CI = 1.171, 2.952]. Compared with urban residents, women in rural settings were more likely to receive results for HTC during ANC [AOR = 1.297; CI = 0.901, 1.867]. Women in the richest wealth quintile were more than five times likely to receive HTC results compared with the poorest women [AOR = 5.565; CI = 2.560, 12.101].

**Table 2:** Binary logistic regression of educational attainment and getting HTC results during ANC.

Variable	Model 1		Model 2	
	COR	95% CI	AOR	95% CI
**Education**				
No Education	1	[1,1]	1	[1,1]
Primary	1.395*	[1.011,1.924]	1.020	[0.686,1.516
Secondary or Higher	2.660***	[1.984,3.568]	1.535*	[1.044,2.258]
**Age**				
15–19			1	[1,1]
20–24			0.916	[0.520,1.613]
25–29			1.220	[0.701,2.122]
30–34			1.693*	[1.171,2.952]
35–39			1.348	[0.792,2.296]
40–44			1.794	[0.828,3.887]
45–49			1.336	[0.469,3.811]
**Residence**				
Urban			1	[1,1]
Rural			1.297	[0.901,1.867]
**Wealth Status**				
Poorest			1	[1,1]
Poorer			1.196	[0.791,1.808]
Middle			1.646*	[1.023,2.648]
Richer			2.877***	[1.636,5.061]
Richest			5.565***	[2.560,12.101]
**ANC Visits**				
Below 4 Visits			1	[1,1]
At least 4 Visits			1.120 *	[1.231,1.649]
**Healthy looking person can have HIV**				
No			1	[1,1]
Yes			0.887	[0.577,1.364]
Don’t Know			0.322**	[0.161,0.646]
**Region of residence**				
Western			1	[1,1]
Central			1.504	[0.726,3.117]
Greater Accra			1.914	[0.966,3.793
Volta			3.379**	[1.547,7.379]
Eastern			1.771	[0.966,3.246]
Ashanti			1.006	[0.556,1.818]
Brong-Ahafo			2.651**	[1.330,5.284]
Northern			0.768	[0.399,1.478]
Upper East			3.139**	[1.499,6.574]
Upper West			1.732	[0.845,3.549]
**Survey Wave**				
2008			1	[1,1]
2014			1.415*	[1.033,1.939]

2008 and 2014 GDHS, COR = Crude Odds Ratio; AOR = Adjusted Odds Ratio CI = Confidence Interval in square brackets; 1 = reference;

*p < 0.05,

**p < 0.01,

***p < 0.001

Women who achieved at least 4 ANC visits were more probable to receive HTC results [AOR = 1.120; CI = 1.231, 1.649] compared to those who had less than 4 ANC visits. Those who did not know whether a healthy-looking person can have HIV or not [AOR = 0.322; CI = 0.161, 0.646] were less likely to receive HTC results compared with women who did not think that a healthy-looking person cannot have HIV. Women of Volta [AOR = 3.379; CI = 1.547, 7.379], Brong Ahafo [2.651; CI = 1.330, 5.284] and Upper East [AOR = 3.139; CI = 1.499, 6.574] regions were more likely to receive HTC results compared to those in the Western region. The odds of receiving HTC results was high in 2014 compared with 2008 [AOR = 1.415; CI = 1.033, 1.939] as illustrated in Table 2.

## Discussion

The focus of this paper was to investigate the influence of the educational attainment of women on receiving HTC results during ANC in Ghana. Our study suggests that educational attainment strongly predicts the tendency for women to receive HTC results during ANC. The findings revealed an increasing trend in receiving HTC results at ANC among women irrespective of educational attainment. This was particularly higher among women with secondary/higher education. Women with secondary/higher education had a higher likelihood to receive HTC results during ANC for the two surveys compared to those with lower or no education and this persisted after controlling for socio-demographic characteristics. There has been an increasing trend in receiving HTC results between 2008 and 2014 with highly educated women on the lead. The increasing trend might indicate adherence to the HTC protocol provided for pregnant women at ANC in an attempt to prevent MTCT or vertical transmission of HIV. This observation is consistent with previous research [12, 13] where educational attainment significantly influenced women’s utilisation of HTC services at ANC.

In highlighting how educational level/status could predict the receipt of HTC results, some studies [14–16] have indicated that, the higher a person’s educational attainment, the more likely the person would fully utilise HTC services. This might imply that educated pregnant women are more likely to understand their perceived vulnerability as explained by Stretcher et al. [17], who have knowledge of MTCT and benefits of HTC thereof, compared to those who have no formal education. In contrast to the current finding, a study by Navaneetham and Dharmalingam [18] showed no association between the educational attainment of women and HTC utilisation during ANC to the latter. In addition to variation in geographical and cultural contexts, the difference might be due to the fact that the current study involved two survey waves and as a result, interventions over the period could have led to respondents receiving their HTC results.

Our findings indicated that women who had at least 4 ANC visits had a high propensity to receive HTC results which agrees with what Muyunda et al. [12] reported using Zambia Demographic and Health Survey data. This could be reasoned that the more a woman attends ANC, the higher her chances of being educated on HTC and understanding its importance, hence aspire to obtain her results. Through ANC, healthcare providers get the opportunity to educate women on a wide range of good practices and HIV tests that need to be conducted to ensure the safety of both the woman and the foetus [19]. Similarly, the 30–34 age category was found to be significantly associated with receiving HTC results at ANC. The probable explanation Kowalczyk et al. [20] gave was the fact that compared to younger women, older women might be more independent in making decisions that affect their health. In addition, it is also possible that older women may be more likely to appreciate the importance of knowing their HIV status compared to younger women and as a result, opt to receive their results. This finding on age and HTC uptake confirms the findings of Sambah et al. [14] who observed a linear progression of age and HTC service utilisation. They explained that as respondents grow in age, they enter into conjugal relationships which possibly predispose them to HIV and other sexually transmitted diseases. Sambah et al. [14] espoused that such exposure may increase HTC service utilisation possibly as a primary preventive tool.

The study revealed that women who were unsure that a healthy-looking person can have HIV were less likely to receive HTC results. Unlike women who are sure that a healthy-looking person cannot have HIV, those who are uncertain may not be curious to receive their test results to clarify their doubts. They may not be eager to know their status upon the least advice from a healthcare practitioner especially during ANC. This is inconsistent with the findings of Udoh and Ushie [21] in Nigeria that women who did not know about HIV (mother to child transmission during pregnancy) were slightly more likely to test for HIV compared with women who knew about mother to child transmission. This is suggestive that the ability of healthcare providers to know what women think and believe about HIV can be explored in packaging HTC advocacy messages.

We found that women from the rural areas, Northern region and those with poorest wealth status were less likely to receive HTC results. These findings could be linked to healthcare access in terms of geographical and financial accessibility. The low receipt of HTC results by rural dwellers, women from the Northern region and the poorest women lend credence to the assertion that poverty and place of residence may deprive and limit rural women from such desired health-seeking behaviours. Also, women in Brong Ahafo, Volta Upper East Region were more likely to receive their HIV test results. This builds on the finding from an earlier study which suggested that differential socio-economic patterns and geographical locations greatly influence HTC uptake [12]. Previous studies [13, 22] observed rural/urban and socioeconomic disparities of respondents to influence their use or non-use of HTC services. The plausible explanation to the high odds of HIV test results receipt in the Brong Ahafo region for instance, could be explained by the upsurge in HIV cases in the region as reported by the GAC [23]. To address the inherent effect of geographical and financial inequities on HTC service utilisation, we suggest that HIV/AIDS prevention models should be incorporated into the non-formal division programmes in rural communities and also, community health nurses need to intensify HTC services as an essential gateway to the prevention of MTCT. Such initiatives can offer relevant messages which could serve as cues to action on the need to undergo HTC during ANC [17].

In interpreting our results, readers should bear in mind that this is a repeated cross-sectional study and therefore precludes causality. The representativeness, consistency and comparable nature of the two surveys utilised to reinforce the generalisability prospects of the study and possible reproducibility in other sub-Saharan African, low and middle-income countries battling with MTCT and the other twenty-two high-priority countries earmarked for PMTCT.

## Conclusions and policy implications

Despite the relatively high receipt of HTC results at ANC observed between 2008 and 2014, our findings revealed disparities driven by educational attainment, wealth status, age, ANC visits and residence. This indicates that women with no education, those from rural areas, younger and poor women are missing out on the full continuum of HTC service at ANC. We suggest the strengthening and equipping of the non-formal division of Ghana Education Service to intensify rural education and literacy especially of the females. Also, the Community Health Nurses and the Community-Based Health Planning and Services (CHPS) concept should be heightened to make it more relevant to rural communities in bridging the disparity between access to quality maternal health services at ANC and health literacy on the benefits of receiving HTC results among others. More so, HTC programmes should be integrated into youth reproductive health programmes at female clubs in schools, women organisations from faith groups (i.e., Churches, Mosques) to the community level tailored to initiate young women into early testing for HIV as a primary and secondary preventive tool.
